# Clinical characteristics and outcomes of *Pseudomonas aeruginosa* bacteremia in febrile neutropenic children and adolescents with the impact of antibiotic resistance: a retrospective study

**DOI:** 10.1186/s12879-017-2597-0

**Published:** 2017-07-17

**Authors:** Hyo Sup Kim, Bo Kyoung Park, Seong koo Kim, Seung Beom Han, Jae Wook Lee, Dong-Gun Lee, Nack-Gyun Chung, Bin Cho, Dae Chul Jeong, Jin Han Kang

**Affiliations:** 1 Department of Pediatrics, Seoul St. Mary’s Hospital, College of Medicine, The Catholic University of Korea, 222 Banpo-daero, Seocho-gu, Seoul, 06591 Republic of Korea; 2 The Catholic Blood and Marrow Transplantation Center, College of Medicine, The Catholic University of Korea, Seoul, Republic of Korea; 3 The Vaccine Bio Research Institute, College of Medicine, The Catholic University of Korea, Seoul, Republic of Korea; 4 Division of Infectious Diseases, Department of Internal Medicine, College of Medicine, The Catholic University of Korea, Seoul, Republic of Korea

**Keywords:** *Pseudomonas aeruginosa*, Antibiotic resistance, Multidrug resistance, Neutropenia, Child

## Abstract

**Background:**

Although the proportion of *Pseudomonas aeruginosa* infections has reduced after the introduction of antibiotics with anti-pseudomonal effects, *P. aeruginosa* bacteremia still causes high mortality in immunocompromised patients. This study determined the clinical characteristics and outcomes of *P. aeruginosa* bacteremia and the antibiotic susceptibilities of strains isolated from febrile neutropenic patients.

**Methods:**

Thirty-one febrile neutropenic children and adolescents with underlying hematologic/oncologic disorders diagnosed with *P. aeruginosa* bacteremia between 2011 and 2016 were enrolled in the study. Their medical records were retrospectively reviewed to evaluate the demographic and clinical characteristics. Antibiotic susceptibility rates of the isolated *P. aeruginosa* to eight antibiotic categories (anti-pseudomonal penicillin, anti-pseudomonal penicillin and β-lactamase inhibitor combination, anti-pseudomonal cephalosporin, monobactam, carbapenem, aminoglycoside, fluoroquinolone, and colistin) were also determined. Among the investigated factors, risk factors for mortality and infections by a multidrug-resistance (MDR) strain were determined.

**Results:**

Thirty-six episodes of *P. aeruginosa* bacteremia were identified. The mean age of the enrolled patients was 9.5 ± 5.4 years, and 26 (72.2%) episodes occurred in boys. Acute myeloid leukemia (41.7%) and acute lymphoblastic leukemia (33.3%) were the most common underlying disorders. The 30-day mortality was 38.9%, and 36.1% of the episodes were caused by MDR strains. The deceased patients were more likely to experience breakthrough infection (*P* = 0.036) and bacteremia (*P* = 0.005) due to MDR strains when compared with the patients who survived. The survived patients more likely received appropriate empirical antibiotic therapy (*P* = 0.024) and anti-pseudomonal β-lactam and aminoglycoside combination therapy (*P* = 0.039) compared with the deceased patients. The antibiotic susceptibility rates of the isolated *P. aeruginosa* strains were as follows: piperacillin/tazobactam, 67.6%; meropenem, 72.2%; and amikacin, 100%.

**Conclusions:**

Mortality due to *P. aeruginosa* bacteremia remained at 38.9% in this study, and more than one-third of the isolated strains were MDR. In this context, empirical antibiotic combination therapy to expand the antibiotic spectrum may be a strategy to reduce mortality due to *P. aeruginosa* bacteremia in febrile neutropenic patients.

## Background

*Pseudomonas aeruginosa* was the most common infectious pathogen in patients with hematologic/oncologic disorders during the 1960s and 1970s; *P. aeruginosa* infections also showed higher mortality rates compared with other bacterial infections [1, 2]. After the introduction of antibiotics with anti-pseudomonal effects in the 1970s and the increased frequency of Gram-positive bacterial infections in the 1980s, the proportion of *P. aeruginosa* infections in immunocompromised patients was reduced. In our hospital, 8.3% of bacteremia episodes diagnosed in children with febrile neutropenia (FN) between 2010 and 2014 were caused by *P. aeruginosa* [3]. However, *P. aeruginosa* is still the third most common Gram-negative cause of bacteremia in FN patients, after *Klebsiella pneumoniae* and *Escherichia coli* [3, 4]. In addition, *P. aeruginosa* bacteremia resulted in approximately 30% mortality in patients with underlying hematologic/oncologic disorders in the 2000s [5–7].

Although multidrug-resistance (MDR) *P. aeruginosa* infections have been increasing since 2000s [4], empirical monotherapy with an anti-pseudomonal β-lactam agent has been recommended for the treatment of FN in patients with underlying hematologic/oncologic disorders [8]. However, the prediction of severe infections due to MDR *P. aeruginosa* strains and an empirical antibiotic combination therapy to broaden the antibiotic spectrum for those patients may improve the prognosis. Nevertheless, the clinical characteristics and outcomes of *P. aeruginosa* bacteremia and risk factors for MDR strain infections have been reported more rarely in children than in adults [7, 9, 10], with only a few recent studies on *P. aeruginosa* bacteremia in FN children [11, 12].

The present study investigated the recent characteristics and outcomes of *P. aeruginosa* bacteremia in FN children and adolescents with underlying hematologic/oncologic disorders and assessed the antibiotic susceptibilities of the *P. aeruginosa* isolates. Risk factors for a grave outcome and infections due to MDR strains were also evaluated.

## Methods

### Patients and study design

Among children and adolescents hospitalized in the Department of Pediatrics of Seoul St. Mary’s Hospital (Seoul, Republic of Korea) between 2011 and 2016, FN children and adolescents <19 years of age with underlying hematologic/oncologic disorders diagnosed with *P. aeruginosa* bacteremia were enrolled in the present study. Seoul St. Mary’s Hospital is a university-affiliated tertiary teaching hospital that has a separate 46-bed ward for children and adolescents with hematologic/oncologic disorders. A mean of 1400 children and adolescents are admitted to the ward and 60–80 allogeneic and autologous hematopoietic cell transplantations are performed annually. This study was performed as a retrospective observational study: the medical records of the enrolled patients were retrospectively reviewed in order to investigate their demographic data, including sex and age. The clinical data included the type and status of the underlying disorders, the therapy administered for treatment of the underlying disorders preceding bacteremia, the presence of focal infections, the type and appropriateness of administered antibiotic agents for FN and bacteremia, and the occurrence of complications and death. In addition, the antibiotic susceptibilities of the isolated *P. aeruginosa* strains were also investigated.

For the whole study population, two comparisons were performed. Firstly, the enrolled patients were divided into survived and deceased groups based on mortality within 30 days after the development of *P. aeruginosa* bacteremia, and a comparison was performed between the two groups in order to identify factors associated with mortality. Secondly, the whole study population was divided into MDR and non-MDR groups based on the antibiotic susceptibilities of the *P. aeruginosa* isolates, and another comparison was performed between the two groups in order to determine the risk factors of MDR strain infections.

### Microbiological tests

Blood samples for the culture studies were collected from a peripheral vein and each lumen of the central venous catheter. Each 1–3 mL blood sample was immediately inoculated into a culture bottle (BD BACTEC™ Peds Plus Culture Vial, Becton Dickinson, Sparks, MD, USA), and transferred to the laboratory. An automated system (BACTEC™ FX, Becton Dickinson) was used for culturing; the bacterial identification and antibiotic susceptibility tests of the *P. aeruginosa* isolates were also performed using an automated system (VITEK®2, bioMériux, Hazelwood, MO, USA). The antibiotics used for the susceptibility tests included piperacillin (anti-pseudomonal penicillin), piperacillin/tazobactam and ticarcillin/clavulanate (anti-pseudomonal penicillin and β-lactamase inhibitor combination), ceftazidime and cefepime (anti-pseudomonal cephalosporin), aztreonam (monobactam), meropenem and imipenem (carbapenem), gentamicin and amikacin (aminoglycoside), ciprofloxacin (fluoroquinolone), and colistin.

### Definitions

Neutropenia was defined as an absolute neutrophil count <500/mm^3^ or an expected absolute neutrophil count <500/mm^3^ within 2 to 3 days on the day when fever developed [13]. Fever was defined as axillary or tympanic membrane temperatures above 37.5 °C or 38.0 °C, respectively [13].

*P. aeruginosa* bacteremia was diagnosed when at least one of the blood sample cultures was positive for *P. aeruginosa*. If *P. aeruginosa* bacteremia was diagnosed within 1 month after the diagnosis of a previous *P. aeruginosa* bacteremia in the same patient, the bacteremia episode was excluded from the present study with an assumption of undertreated previous bacteremia. Polymicrobial infection was defined as the presence of bacteria other than *P. aeruginosa* identified from blood samples collected on the same day or as other viral or fungal infections identified during the bacteremia period. Serum galactomannan levels were measured twice a week during each neutropenic period, and a multiplex polymerase chain reaction assay for respiratory viruses was performed in patients with respiratory symptoms. For patients complaining of diarrhea during antibiotic therapy, a *Clostridium difficile* toxin assay was performed. Breakthrough infection was defined as the diagnosis of *P. aeruginosa* bacteremia in a patient who had been receiving antibiotic agents with anti-pseudomonal effects for more than 2 days. Empirical antibiotic therapy was considered appropriate if the identified *P. aeruginosa* strain was susceptible to at least one of the empirical antibiotic agents administered within 24 h of the development of FN.

The presence of focal infections was determined by two independent pediatricians based on patients’ symptoms, physical examination and radiological findings. Complications due to bacteremia included shock, hypoxia, mechanical ventilation, and renal and hepatic insufficiencies. Shock was defined when the patient showed systolic blood pressure < 5th percentile for an age-matched normal range despite fluid resuscitation or received inotropic agents to maintain blood pressure [14]. Hypoxia was defined when oxygen supplementation was performed to maintain a SpO_2_ > 90%. Renal insufficiency was defined as serum creatinine levels more than twice those from before bacteremia [15]. Hepatic insufficiency was defined as serum aspartate transaminase or alanine transaminase levels more than twice those from before bacteremia, with a serum total bilirubin ≥2.0 mg/dL and prothrombin time international normalized ratio ≥ 1.5 [16]. All patients who died within 30 days after the development of bacteremia were included in the deceased group. The patients who died of uncontrolled focal complications of *P. aeruginosa* bacteremia were also included in the deceased group regardless of the time of death.

The antibiotic susceptibility was determined based on the Clinical and Laboratory Standards Institute 2010 recommendations. Among the automated antibiotic susceptibility test results, ‘intermediate’ and ‘resistance’ were categorized as non-susceptible. Although isepamicin was the most frequently administered aminoglycoside in the enrolled patients, susceptibility tests for isepamicin were not performed in our hospital. Therefore, antibiotic susceptibility to isepamicin was determined based on the results for amikacin. MDR was defined as *P. aeruginosa* strains resistant to three or more of the eight recommended antibiotic categories to be tested [17].

### Statistical analysis

In the comparisons between patient groups, continuous variables were compared using a Student’s t-test or a Mann-Whitney test based on their normal distributions, and categorical variables were compared using chi-square tests. Statistical analyses were performed using IBM SPSS Statistics for Windows, version 21.0 (IBM Corporation, Armonk, NY, USA), with the statistical significance defined as a two-tailed *P* value <0.05.

## Results

### Characteristics of patients diagnosed with *P. aeruginosa* bacteremia

A total of 36 episodes of *P. aeruginosa* bacteremia were diagnosed in the 31 FN children and adolescents during the study period. Among them, three patients each experienced two episodes and one patient experienced three episodes of *P. aeruginosa* bacteremia. The recurrent episodes occurred a median of 7 weeks (range 4–33 weeks) after the previous *P. aeruginosa* bacteremia.

The mean age of the enrolled patients was 9.5 ± 5.4 years, and 26 (72.2%) episodes occurred in boys (Table 1). Acute myeloid leukemia (15, 41.7%) and acute lymphoblastic leukemia (12, 33.3%) were most common underlying disorders. Among 30 patients with underlying malignancies, except for five patients with severe aplastic anemia (SAA) and one patient with severe combined immune deficiency, only eight (26.7%) patients were in the complete remission or response of their underlying malignancies. Accordingly, re-induction (13, 36.1%) and palliative (seven, 19.4%) chemotherapy were the most frequently administered therapies preceding bacteremia. Three (8.3%) episodes of bacteremia occurred in patients with SAA who had not received any immune suppression therapy or hematopoietic cell transplantations. Polymicrobial infections were diagnosed in nine (25.0%) episodes: three (33.3%) of invasive pulmonary aspergillosis (IPA), two (22.2%) of *E. coli* bacteremia, and one each (11.1%) of *Enterobacter cloacae*, methicillin-susceptible *Staphylococcus aureus*, methicillin-resistant coagulase-negative staphylococci bacteremia and *C. difficile*-associated diarrhea. Breakthrough infections were identified in eight (22.2%) episodes. Among them, seven (87.5%) episodes occurred in meropenem therapy, and one (12.5%) occurred in piperacillin/tazobactam therapy. Focal infections accompanied 26 (72.2%) episodes, most frequently as gastroenteritis (15, 41.7%) and respiratory tract infections (11, 30.6%). Piperacillin/tazobactam and isepamicin combination therapy (16, 44.4%) were most commonly administered as empirical antibiotic therapy. Empirical antibiotic therapy was appropriate for 30 episodes (83.3%); however, empirically administered β-lactam agents were appropriate for 24 episodes (66.7%). Complications occurred in 17 (47.2%) episodes; hypoxia (14, 38.9%) and shock (13, 36.1%) were most common. Fourteen (38.9%) patients died and were included in the deceased group.

**Table 1:** Characteristics of febrile neutropenic children and adolescents with *Pseudomonas aeruginosa* bacteremia

Factor	Number (%)
Male sex	26 (72.2)
Age (years), mean ± SD	9.5 ± 5.4
Underlying disorders	
Acute myeloid leukemia Acute lymphoblastic leukemia Severe aplastic anemia Neuroblastoma Lymphoma Severe combined immune deficiency	15 (41.7) 12 (33.3) 5 (13.9) 2 (5.6) 1 (2.8) 1 (2.8)
Remission state of underlying malignancy^a^	
Complete remission Non-complete remission	8 (26.7) 22 (73.3)
Administered therapy preceding bacteremia	
Induction chemotherapy Re-induction chemotherapy Consolidation chemotherapy Autologous hematopoietic cell transplantation Allogeneic hematopoietic cell transplantation Palliative chemotherapy None^b^	2 (5.6) 13 (36.1) 6 (16.7) 1 (2.8) 4 (11.1) 7 (19.4) 3 (8.3)
Central venous catheter	
Hickman catheter Subcutaneously implanted chemoport None	27 (75.0) 7 (19.4) 2 (5.6)
Polymicrobial infection	9 (25.0)
Breakthrough infection	8 (22.2)
Local infection Gastrointestinal tract infection Respiratory tract infection Skin and soft tissue infection Catheter site infection	26 (72.2) 15 (41.7) 11 (30.6) 7 (19.4) 2 (5.6)
Empirical antibiotic therapy	
Piperacillin/tazobactam with aminoglycoside Meropenem Cefepime Cefepime with aminoglycoside Meropenem with aminoglycoside	16 (44.4) 14 (38.9) 3 (8.3) 2 (5.6) 1 (2.8)
Empirical combination antibiotic therapy	19 (52.8)
Appropriateness of empirical antibiotics	
Overall β-lactam agents	30 (83.3) 24 (66.7)
Fever duration (days), median (range)	4 (1–53)
Complications Hypoxia Shock Mechanical ventilator care Renal dysfunction Hepatic dysfunction	17 (47.2) 14 (38.9) 13 (36.1) 8 (22.2) 8 (22.2) 4 (11.1)
Death	14 (38.9)
Multidrug-resistant strain infections	13 (36.1)

*SD*, standard deviation

^a^Remission state of the underlying malignancy was determined in 30 children except those with non-malignant underlying disorders

^b^Three children with severe aplastic anemia had not received any therapy prior to the development of bacteremia

**Fig. 1:**
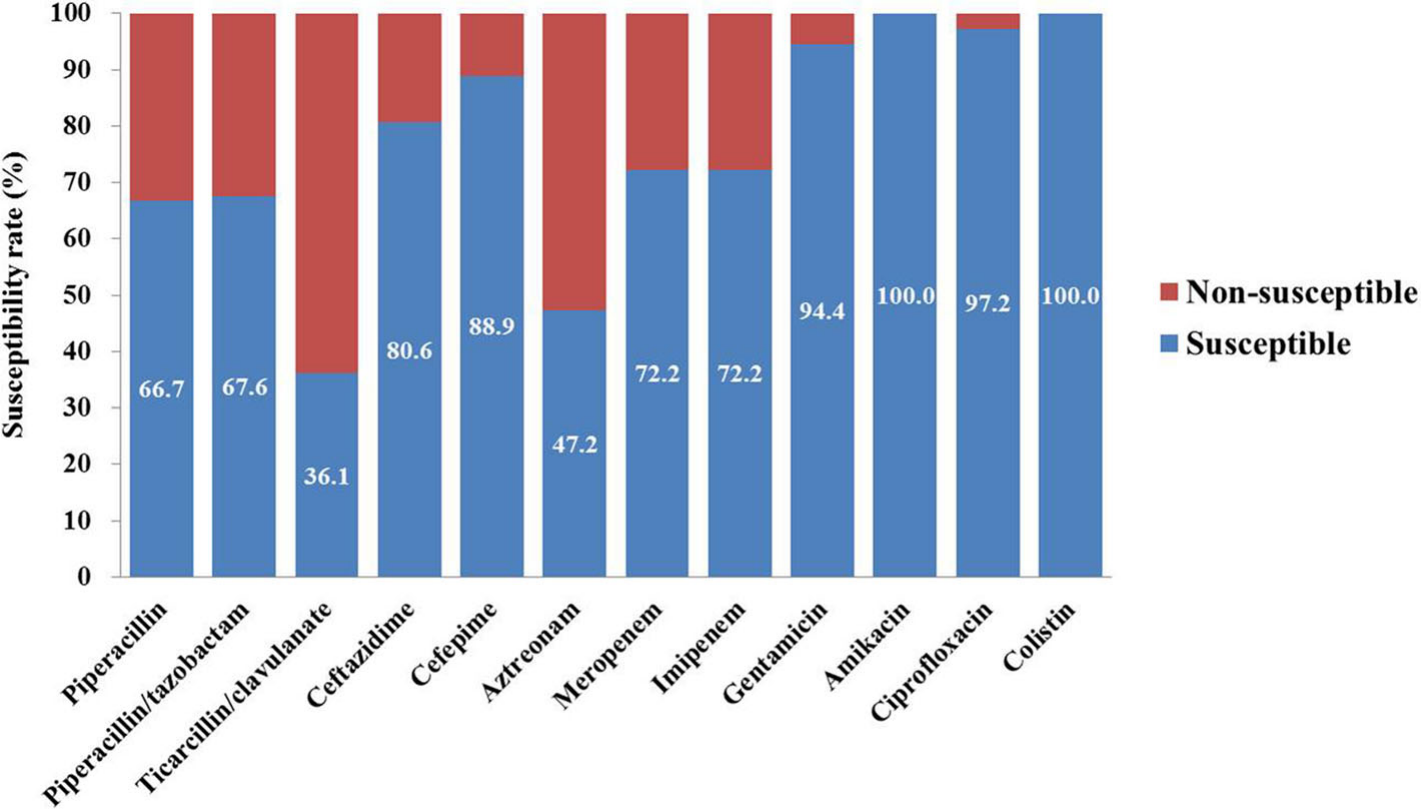
Antibiotic susceptibility rates of the isolated *P. aeruginosa* strains. Anti-pseudomonal penicillin and β-lactamase inhibitor combination agents and anti-pseudomonal cephalosporins, which are recommended as empirical antibiotic agents for neutropenic febrile patients showed variable susceptibility rates (36.1% ~ 88.9%). Amikacin and colistin showed the highest susceptibility rates, whereas, carbapenems showed a susceptibility rate of 72.2%

The antibiotic susceptibility rates to amikacin, colistin, and ciprofloxacin were 100%, 100%, and 97.2%, respectively (Fig. 1). Piperacillin/tazobactam and cefepime, which have been most frequently used in our hospital as empirical antibiotics for FN patients, were effective against 67.6% and 88.9% of the *P. aeruginosa* isolates, respectively. The carbapenem susceptibility rate was 72.2%. MDR strains were identified in 13 (36.1%) episodes (Table 1). No strains were pandrug-resistant; however, three (8.3%) strains showed extensive drug resistance, in which they were susceptible to only one or two categories of antibiotics.

### Clinical factors associated with mortality

Fourteen (38.9%) patients died a median of 5 days (range 0–43 days) after the development of bacteremia. Four (28.6%) patients died despite the resolution of *P. aeruginosa* bacteremia. Among these four patients, two died of uncontrolled IPA, and one died of uncontrolled relapse of acute leukemia. The remaining child died of uncontrolled intra-abdominal infection 43 days after the development of *P. aeruginosa* bacteremia.

The deceased patients experienced significantly more breakthrough infections compared with those of the survived group (*P* = 0.036, Table 2). In the survived group, two (9.1%) patients experienced breakthrough infections in meropenem therapy caused by meropenem-sensitive and meropenem-resistant strains, respectively. Six (42.9%) patients in the deceased group experienced breakthrough infections. Four (66.7%) were during meropenem therapy, which had continued until the antibiotic susceptibility test findings were reported, and were infected with meropenem-resistant strains. Another deceased patient infected with a meropenem-resistant strain was also receiving meropenem therapy, and isepamicin was added on the day of fever. One deceased patient was receiving piperacillin/tazobactam therapy, which was changed to meropenem on the first day of fever; however, a meropenem-resistant strain was identified.

**Table 2:** Comparison of characteristics between the survived and deceased groups

Factor	Survived group (*n* = 22)	Deceased group (*n* = 14)	*P* value
Male sex	13 (59.1)	13 (92.9)	0.054
Age (years), mean ± SD	9.2 ± 5.3	10.0 ± 5.7	0.663
Underlying disorders			0.294
Acute myeloid leukemia Acute lymphoblastic leukemia Severe aplastic anemia Neuroblastoma Lymphoma Severe combined immune deficiency	12 (54.5) 6 (27.3) 3 (13.6) 1 (4.5) 0 (0.0) 0 (0.0)	3 (21.4) 6 (42.9) 2 (14.3) 1 (7.1) 1 (7.1) 1 (7.1)	
Remission state of underlying malignancy^a^			0.199
Complete remission Non-complete remission	7 (36.8) 12 (63.2)	1 (9.1) 10 (90.9)	
Administered therapy preceding bacteremia			0.123
Induction chemotherapy Re-induction chemotherapy Consolidation chemotherapy Autologous hematopoietic cell transplantation Allogeneic hematopoietic cell transplantation Palliative chemotherapy None^b^	2 (9.1) 8 (36.4) 6 (27.3) 0 (0.0) 2 (9.1) 2 (9.1) 2 (9.1)	0 (0.0) 5 (35.7) 0 (0.0) 1 (7.1) 2 (14.3) 5 (35.7) 1 (7.1)	
Central venous catheter			0.318
Hickman catheter Subcutaneously implanted chemoport None	17 (77.3) 3 (13.6) 2 (9.1)	10 (71.4) 4 (28.6) 0 (0.0)	
Polymicrobial infection	4 (18.2)	5 (35.7)	0.267
Breakthrough infection	2 (9.1)	6 (42.9)	0.036
Local infection	15 (68.2)	11 (78.6)	0.706
Gastrointestinal tract infection Respiratory tract infection Skin and soft tissue infection Catheter site infection	9 (40.9) 4 (18.2) 5 (22.7) 2 (9.1)	6 (42.9) 7 (50.0) 2 (14.3) 0 (0.0)	0.908 0.067 0.681 0.511
Empirical antibiotic therapy			0.008
Piperacillin/tazobactam with aminoglycoside Meropenem Cefepime Cefepime with aminoglycoside Meropenem with aminoglycoside	13 (59.1) 4 (18.2) 3 (13.6) 2 (9.1) 0 (0.0)	3 (21.4) 10 (71.4) 0 (0.0) 0 (0.0) 1 (7.1)	
Empirical combination antibiotic therapy	15 (68.2)	4 (28.6)	0.039
Appropriateness of empirical antibiotics			
Overall β-lactam agents	21 (95.5) 16 (72.7)	9 (64.3) 8 (57.1)	0.024 0.471
Fever duration (days), median (range)	2 (1–53)	4 (1–14)	0.713
Complications Hypoxia Shock Mechanical ventilator care Renal dysfunction Hepatic dysfunction	4 (18.2) 2 (9.1) 3 (13.6) 2 (9.1) 1 (4.5) 0 (0.0)	13 (92.9) 12 (85.7) 10 (71.4) 6 (42.9) 7 (50.0) 4 (28.6)	<0.001 <0.001 <0.001 0.036 0.003 0.017
Multidrug-resistant strain infections	4 (18.2)	9 (64.3)	0.005

*SD,* standard deviation

^a^Remission state of underlying malignancy was determined in 30 children except those with non-malignant underlying disorders

^b^Three children with severe aplastic anemia had not received any therapy prior to the development of bacteremia

Empirical antibiotic combination therapy was administered more frequently in the survived group than in the deceased group (*P* = 0.039). Empirical antibiotic therapy was appropriate for 95.5% and 64.3% of cases in the survived and deceased groups, respectively (*P* = 0.024); however, the appropriateness of the empirically administered β-lactam agents, except for combined aminoglycosides, did not differ significantly between the two groups.

Infections due to MDR strains were significantly more frequent in the deceased group than in the survived group (*P* = 0.005). Among the tested antibiotics, the susceptibility rates of piperacillin (86.4% vs. 35.7%, *P* = 0.003), aztreonam (68.2% vs. 14.3%, *P* = 0.002), and carbapenems (86.4% vs. 50.0%, *P* = 0.026) were significantly lower in the deceased group than in the survived group.

### Clinical factors associated with MDR *P. aeruginosa* infections

Significantly more patients in the MDR group experienced breakthrough infections (*P* < 0.001) and had a Hickman catheter (*P* = 0.034, Table 3) compared with those in the non-MDR group. Although the type and frequency of combination empirical antibiotic agents did not differ significantly between the two groups, the appropriateness of empirical antibiotics was significantly lower in the MDR group than in the non-MDR group (*P* = 0.001). The occurrence rate of complications was not significantly different between the two groups; however, mortality was significantly higher in the MDR group than in the non-MDR group (*P* = 0.005).

**Table 3:** Comparison of characteristics between the MDR and non-MDR groups

Factor	Non-MDR group (*n* = 23)	MDR group (*n* = 13)	*P* value
Male sex	14 (60.9)	12 (92.3)	0.060
Age (years), mean ± SD	9.4 ± 5.7	9.8 ± 5.0	0.825
Underlying disorders			0.389
Acute myeloid leukemia Acute lymphoblastic leukemia Severe aplastic anemia Neuroblastoma Lymphoma Severe combined immune deficiency	8 (34.8) 10 (43.5) 3 (13.0) 1 (4.3) 1 (4.3) 0 (0.0)	7 (53.8) 2 (15.4) 2 (15.4) 1 (7.7) 0 (0.0) 1 (7.7)	
Remission state of underlying malignancy^a^			0.682
Complete remission Non-complete remission	6 (30.0) 14 (70.0)	2 (20.0) 8 (80.0)	
Administered therapy preceding bacteremia			0.454
Induction chemotherapy Re-induction chemotherapy Consolidation chemotherapy Autologous hematopoietic cell transplantation Allogeneic hematopoietic cell transplantation Palliative chemotherapy None^b^	2 (8.7) 9 (39.1) 5 (21.7) 0 (0.0) 2 (8.7) 3 (13.0) 2 (8.7)	0 (0.0) 4 (30.8) 1 (7.7) 1 (7.7) 2 (15.4) 4 (30.8) 1 (7.7)	
Central venous catheter			0.034
Hickman catheter Subcutaneously implanted chemoport None	14 (60.9) 7 (30.4) 2 (8.7)	13 (100.0) 0 (0.0) 0 (0.0)	
Polymicrobial infection	7 (30.4)	2 (15.4)	0.438
Breakthrough infection	1 (4.3)	7 (53.8)	<0.001
Local infection Gastrointestinal tract infection Respiratory tract infection Skin and soft tissue infection Catheter site infection	19 (82.6) 9 (39.1) 8 (34.8) 6 (26.1) 2 (8.7)	7 (53.8) 6 (46.2) 3 (23.1) 1 (7.7) 0 (0.0)	0.119 0.681 0.708 0.382 0.525
Previous antibiotic therapy	18 (78.3)	12 (92.3)	0.385
Empirical antibiotic therapy			0.078
Piperacillin/tazobactam with aminoglycoside Meropenem Cefepime Cefepime with aminoglycoside Meropenem with aminoglycoside	13 (56.5) 6 (26.1) 3 (13.0) 1 (4.3) 0 (0.0)	3 (23.1) 8 (61.5) 0 (0.0) 1 (7.7) 1 (7.7)	
Empirical combination antibiotic therapy	14 (60.9)	5 (38.5)	0.196
Appropriateness of empirical antibiotics			
Overall β-lactam agents	23 (100.0) 21 (91.3)	7 (53.8) 3 (23.1)	0.001 <0.001
Fever duration (days), median (range)	3 (1–53)	4 (1–32)	0.745
Complications Hypoxia Shock Mechanical ventilator care Renal dysfunction Hepatic dysfunction	9 (39.1) 7 (30.4) 7 (30.4) 5 (21.7) 4 (17.4) 2 (8.7)	8 (61.5) 7 (53.8) 6 (46.2) 3 (23.1) 4 (30.8) 2 (15.4)	0.299 0.166 0.474 1.000 0.422 0.609
Mortality	5 (21.7)	9 (69.2)	0.005

*MDR,* multidrug-resistant; *SD,* standard deviation

^a^Remission state of underlying malignancy was determined in 30 children except those with non-malignant underlying disorders

^b^Three children with severe aplastic anemia had not received any therapy prior to the development of bacteremia

## Discussion

The present study investigated the clinical characteristics and outcomes of *P. aeruginosa* bacteremia in FN children and adolescents. Mortality due to *P. aeruginosa* bacteremia remained high in the 2010s, and more than one-third of the isolated *P. aeruginosa* strains were MDR.

The mortality among immunocompromised patients with *P. aeruginosa* bacteremia was approximately 70% in the 1960s and 1970s [1, 2, 18], which decreased to 20–25% in the 1990s with the use of anti-pseudomonal antibiotics [18, 19]. However, the mortality in the 2000s was 20–39%, similar to that in the 1990s [5–7, 11], and 38.9% of FN children and adolescents with *P. aeruginosa* bacteremia died in the present study. This recent slowdown in improving outcomes in *P. aeruginosa* bacteremia patients might be associated with increasing prevalence of antibiotic-resistant strains. MDR *P. aeruginosa* comprised 1.6–8.2% of the identified *P. aeruginosa* strains until the early 2000s [20, 21]; however, the proportion of MDR strains increased to 30.7–71.1% in the late 2010s [5, 6, 11]. In Korea, 11.3% of *P. aeruginosa* bacteremia cases diagnosed in hospitalized children, including immune-competent and -compromised children, were caused by MDR strains in the 2000s [10]; however, 36.1% of *P. aeruginosa* bacteremia were caused by MDR strains in the present study.

The appropriateness of empirical antibiotic therapy as well as infection due to MDR strains was associated with mortality in patients with *P. aeruginosa* bacteremia, a relationship that has been previously reported [5–7, 18, 22–25]. In sum, antibiotics to which MDR *P. aeruginosa* strains are susceptible should be administered empirically in order to improve the outcomes of immunocompromised patients with *P. aeruginosa* bacteremia. The *P. aeruginosa* antibiotic susceptibility rates in the present study were 100% to aminoglycosides and colistin and 97.2% to ciprofloxacin, which were higher than those to anti-pseudomonal β-lactam agents such as piperacillin/tazobactam and cefepime. Previous studies on *P. aeruginosa* bacteremia in children also reported higher antibiotic susceptibility rates to amikacin and fluoroquinolones compared with those of β-lactam agents [10, 11]. However, the use of fluoroquinolones has been restricted in children due to concerns of skeletal adverse effects, and empirical use of colistin may not be appropriate considering its nephrotoxicity and neurotoxicity [26]. Aminoglycosides are not effective as a single agent against Gram-negative bacterial infections including pseudomonal infections [22, 24, 27, 28]. As a result, anti-pseudomonal β-lactam agent and aminoglycoside combination therapy may be helpful to broaden the antibiotic coverage for MDR strains and consequently improve the outcomes of patients with *P. aeruginosa* bacteremia. In the present study, although the appropriateness of the empirical β-lactam agents did not differ significantly between the survived and deceased groups, the combination with aminoglycosides significantly increased the appropriateness of the empirical antibiotics in the survived group. However, the contribution of the β-lactam agent and aminoglycoside combination to antibiotic synergism, improved clinical outcomes, and suppressed the emergence of antibiotic resistance has not been confirmed [29–31]. Therefore, this antibiotic combination can be maintained for early (3 to 5 days) bacteremia, followed by targeted antibiotic therapy based on the antibiotic susceptibility results [23].

The relationship between infections due to MDR strains and mortality of patients with *P. aeruginosa* bacteremia in the present study underscore the need to decrease the prevalence of MDR strains. Infections due to MDR *P. aeruginosa* were associated with recent use of carbapenems, ventilator care, and *P. aeruginosa* infection or colonization within the previous year [32, 33]. In Korean children, the primary risk factor for MDR *P. aeruginosa* bacteremia was admission to the intensive care unit within 1 month [10]; however, no patient in the present study had been admitted to the intensive care unit within 2 months before developing *P. aeruginosa* bacteremia. Almost all patients in the present study had received repeated anti-pseudomonal antibiotic therapy due to their underlying hematologic/oncologic disorders; therefore, recent use of anti-pseudomonal antibiotics was not significantly associated with MDR strain infections. However, the effect of recent antibiotic use on the MDR strain infections cannot be ignored, considering the relationship between breakthrough and MDR strain infections. Previous studies have reported that various β-lactam agents and fluoroquinolones were related to MDR *P. aeruginosa* infections [20, 30, 34]. In addition, the induction rate of antibiotic resistance in *P. aeruginosa* was affected by the type of previously administered antibiotics, and imipenem showed a higher rate of resistance induction compared with those of other antibiotic agents [30]. Accordingly, restriction of carbapenem use may reduce the emergence of MDR *P. aertuginosa* strains. Carbapenems have an additional antibiotic effect against extended-spectrum β-lactamase (ESBL)-producing Enterobacteriaceae beyond other anti-pseudomonal β-lactam agents commonly used for FN patients. Our previous study, however, showed that a combination of empirical β-lactam agent and aminoglycoside instead of carbapenems did not cause unfavorable outcomes in FN patients with ESBL-producing *E. coli* and *K. pneumoniae* infections [35]. As a result, empirical anti-pseudomonal β-lactam and aminoglycoside combination therapy in FN patients may reduce carbapenem use and subsequently prevent the emergence of antibiotic resistance without worsening prognosis due to Gram-negative bacterial infections. In our hospital, anti-pseudomonal β-lactam and aminoglycoside combination therapy has been used as first-line empirical therapy for FN patients. However, carbapenems have been administered as a second-line empirical antibiotic agent for patients with persistent fever despite the first-line empirical antibiotic therapy until the recovery of neutropenia. Eventually, many patients might have received carbapenems for longer days than anti-pseudomonal penicillins or anti-pseudomonal cephalosporins during their hospitalization. Such prolonged use of carbapenems might cause the emergence of MDR *P. aeruginosa* strains in our hospital, and therefore, further efforts to shorten the duration of empirical carbapenem use should be performed.

The present study had several limitations. First, *P. aeruginosa* comprise about 10% of the pathogens identified in FN patients; thus, the number of FN patients with *P. aeruginosa* bacteremia was small. The increase in the number of enrolled patients may reveal additional factors related to mortality and MDR strain infections. A multicenter study is necessary to overcome this limitation; however, each hospital may have their own strategies for chemotherapy, transplantation, and antibiotic therapy in FN patients. In addition, the antibiotic resistance patterns of each hospital reflect the resistance patterns of individual communities and countries [21]. Therefore, interpretation of the results of a multicenter or multinational study on the antibiotic susceptibilities may be difficult. Also, this study was a retrospective observational study, and therefore, a well-designed prospective cohort study or case-control study is necessary to overcome such limitations. Second, the appropriateness of targeted antibiotic therapy rather than empirical therapy can affect the outcomes of patients with bacteremia. In the present study, we could not evaluate the effect of targeted therapy on the outcomes because 14 types of targeted antibiotic therapy were performed in 36 episodes.

## Conclusions

In conclusion, *P. aeruginosa* bacteremia in FN children and adolescents exhibited continued high mortality in the 2010s, and MDR strain infections occurred more frequently than before. Mortality in patients with *P. aeruginosa* bacteremia was associated with MDR strain infections and the appropriateness of empirical antibiotic therapy. Therefore, ongoing surveillance for MDR *P. aeruginosa* infections and efforts to reduce MDR strains are necessary. In addition, anti-pseudomonal β-lactam agent and aminoglycoside combination therapy may be useful for empirical antibiotic therapy in FN patients to improve the appropriateness of empirical antibiotics.

## Abbreviations

ESBL: Extended-spectrum β-lactamase
FN: Febrile neutropenia
IPA: Invasive pulmonary aspergillosis
MDR: Multidrug-resistance
SAA: Severe aplastic anemia
